# Sequence variations, flanking region mutations, and allele frequency at 31 autosomal STRs in the central Indian population by next generation sequencing (NGS)

**DOI:** 10.1038/s41598-021-02690-5

**Published:** 2021-12-01

**Authors:** Hirak Ranjan Dash, Kamlesh Kaitholia, R. K. Kumawat, Anil Kumar Singh, Pankaj Shrivastava, Gyaneshwer Chaubey, Surajit Das

**Affiliations:** 1 DNA Fingerprinting Unit, Integrated High-Tech Complex, Forensic Science Laboratory, Bhopal, Madhya Pradesh 462003 India; 2 DNA Division, State Forensic Science Laboratory, Jaipur, Rajasthan 302016 India; 3 DNA Fingerprinting Unit, State Forensic Science Laboratory, Sagar, Madhya Pradesh 769001 India; 4 Cytogenetics Laboratory, Department of Zoology, Banaras Hindu University, Varanasi, 221005 India; 5 Department of Life Science, National Institute of Technology, Rourkela, Odisha 470001 India

**Keywords:** Biotechnology, Genetics, Molecular biology

## Abstract

Capillary electrophoresis-based analysis does not reflect the exact allele number variation at the STR loci due to the non-availability of the data on sequence variation in the repeat region and the SNPs in flanking regions. Herein, this study reports the length-based and sequence-based allelic data of 138 central Indian individuals at 31 autosomal STR loci by NGS. The sequence data at each allele was compared to the reference hg19 sequence. The length-based allelic results were found in concordance with the CE-based results. 20 out of 31 autosomal STR loci showed an increase in the number of alleles by the presence of sequence variation and/or SNPs in the flanking regions. The highest gain in the heterozygosity and allele numbers was observed in D5S2800, D1S1656, D16S539, D5S818, and vWA. rs25768 (A/G) at D5S818 was found to be the most frequent SNP in the studied population. Allele no. 15 of D3S1358, allele no. 19 of D2S1338, and allele no. 22 of D12S391 showed 5 isoalleles each with the same size and with different intervening sequences. Length-based determination of the alleles showed Penta E to be the most useful marker in the central Indian population among 31 STRs studied; however, sequence-based analysis advocated D2S1338 to be the most useful marker in terms of various forensic parameters. Population genetics analysis showed a shared genetic ancestry of the studied population with other Indian populations. This first-ever study to the best of our knowledge on sequence-based STR analysis in the central Indian population is expected to prove the use of NGS in forensic case-work and in forensic DNA laboratories.

## Introduction

Exploration of the targeted STRs using capillary electrophoresis (CE) has been currently considered as the gold standard technology in the forensic DNA analysis. This technology employs the polymerase chain reaction (PCR) followed by CE for the detection of individual-specific length variations at the STR markers. Despite many advantages, CE technology is inexpedient in the analysis of multiple genetic polymorphisms (STRs/SNPs) in a single reaction, simultaneous generation of sequence information of STR alleles, loss of a generation of valuable genetic information from the degraded samples, and generation of low-resolution results in mtDNA and mixture analysis^1^. Since the CE technology does not provide the information on the base-pair variations at the STR alleles, it underestimates the genetic diversity and variations present at that genetic locus. Besides, homoplasmy i.e., similar-sized DNA fragments with varied sequence compositions can be misinterpreted as homozygous due to the generation of a single peak in CE results^2^.

In context to abovesaid drawbacks associated with CE, Next-generation sequencing (NGS) appears to be a suitable alternative technique. It provides information from numerous STRs and SNPs simultaneously. Sequencing of STR alleles provides in-depth genetic information in terms of internal sequence variation and mutations in the samples. NGS is also useful in mtDNA sequencing which is expedient in degraded samples due to the presence of thousands of copies of mtDNA per cell^3^. In addition to the use in routine forensic identification, the NGS technology promises many other applications of forensic relevance such as age estimation^4^, body fluid identification^5^, forensic genealogy^6^, DNA phenotyping^7^, detection of geographic origin and ancestry of an individual^7^. The use of NGS technology decreases the probability of false-positive matches in the DNA profiling due to high resolution in distinguishing between DNA mixtures^8^. Based on these merits, the technology has been considered as the future of forensic DNA analysis.

The major advantage of NGS over CE technology is that there exists no limitation in the number of STR markers to be multiplexed in a single reaction. Therefore, many new STR marker sets have been included in the commercially available sequencing kits besides the recommended 20 core CODIS STR loci. However, before their forensic application, these loci and their aptness at the population level should be understood utterly. The inclusion of more markers could increase the discrimination power of a multiplex system. However, a limited number of genetic markers can be accommodated in a single multiplex reaction due to the involvement of different dye sets and limited channels for detection. This could be overcome by NGS analysis where numerous genetic markers can be analyzed simultaneously.

**Figure 1:**
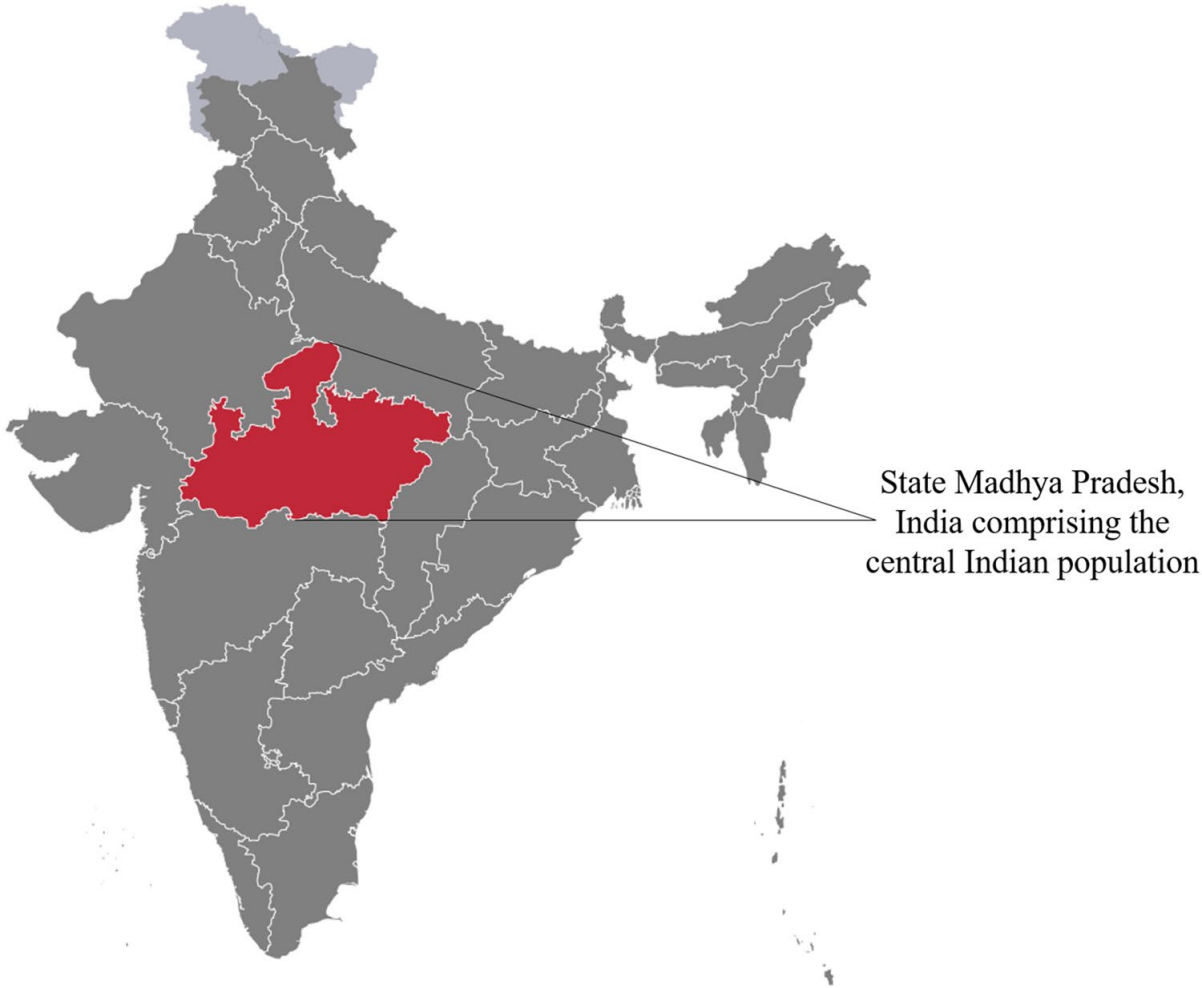
Map of India highlighting the central Indian population residing in the state Madhya Pradesh, India (https://en.wikipedia.org/wiki/Madhya_Pradesh#/media/File:IN-MP.svg).

Several attempts have been made to assess the sequence-based allele frequency data for the autosomal STR markers. Most of the studies such as for the US population^9^, Native Americans from West-Central Arizona^10^, Yavapai native Americans^11^, White British and British Chinese populations^12^ and Danish population^13^ have used ForenSeq DNA Signature Prep Kit on a MiSeq FGx instrument (Illumina, San Diego, CA). On the contrary, limited studies are available for sequence-based allele data using Precision ID Global Filer™ NGS STR panel (Thermo Scientific, US) for the Spanish population^14^ and Han population^15^. Indian population has not yet been explored for their sequence-based STR allelic data. Therefore, an attempt was made in the present study to analyze 31 autosomal STR markers simultaneously i.e., D12S391, D13S317, D8S1179, D21S11, D3S1358, D5S818, D1S1656, D2S1338, vWA, D2S441, D5S2800, D7S820, D16S539, D6S474, D12ATA63, D4S2408, D6S1043, D19S433, D14S1434, CSF1PO, D10S1248, D18S51, D1S1677, D22S1045, D2S1776, D3S4529, FGA, Penta D, Penta E, TH01 and TPOX in the central Indian population (Fig. 1). Madhya Pradesh is the second largest geographical state of India and the fifth largest in terms of population. Being located in the middle of India, Madhya Pradesh shares its boundary with five other states including Uttar Pradesh in North, Chhattisgarh in East, Maharashtra in south, and Gujarat and Rajasthan in West. For this region, the state experiences an admixed of populations to represent mini-India. Understanding the genetic diversity of central Indian population gives a representation of the genetic print pan-India. The study aimed to generate sequence-based allele frequency data, population-specific characteristics, sequence variations, and SNPs in the flanking regions for the forensic casework applications in the studied population.

## Results and discussion

### Sequencing performance of precision ID NGS STR panel v2

**Figure 2:**
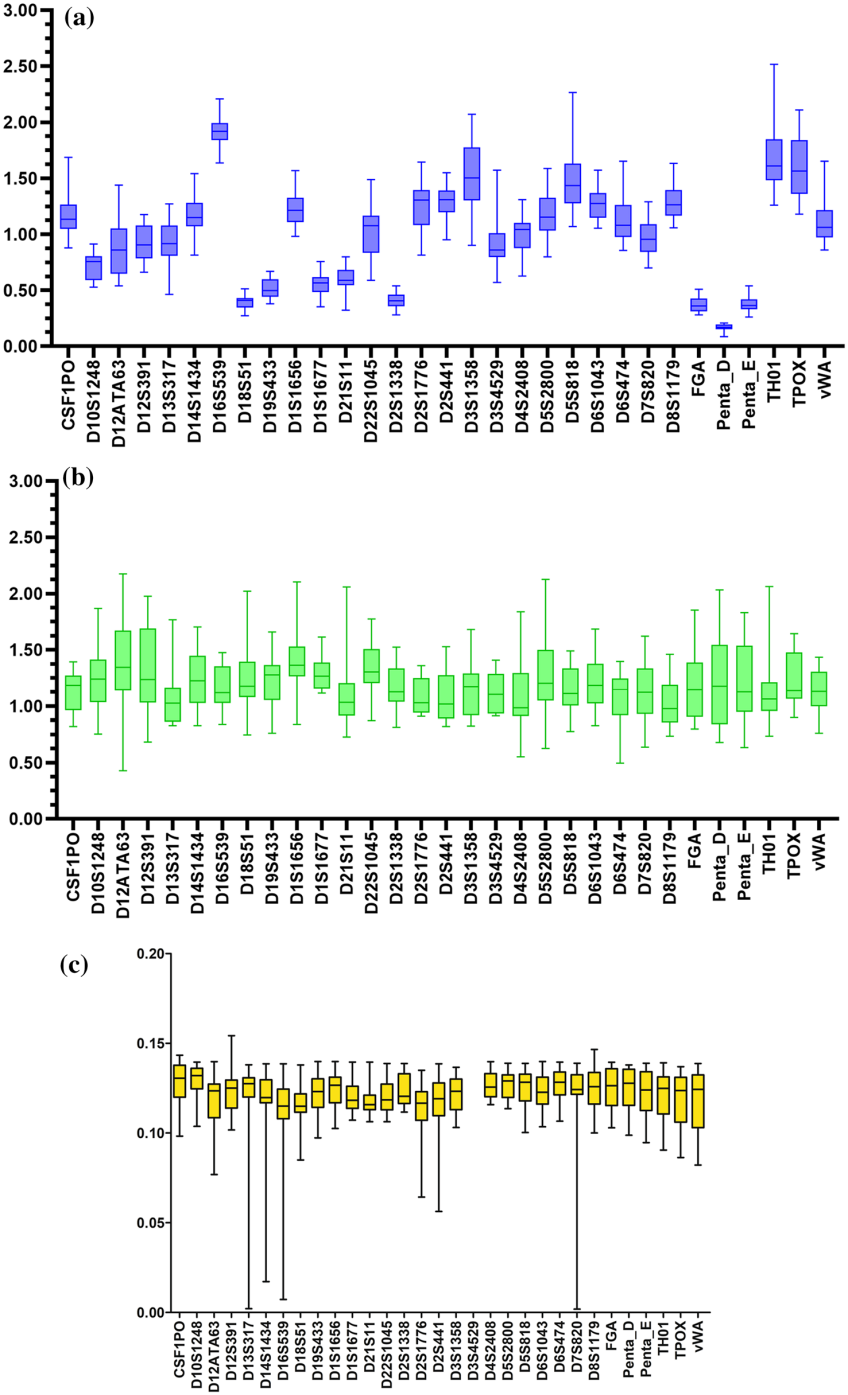
Sequence performance of Precision ID NGS STR panel v2. (**a**) Locus Balance for all STRs measured as coverage of each locus divided by average coverage of all locus per sample. (**b**) Heterozygous Balance for all STRs measured as coverage ratio of one allele to another, for heterozygous genotypes only. (**c**) Stutter ratio measured as ratio of coverage of stutter peak and allele peak.

Quality control parameters such as Locus balance (LB), Heterozygous balance (HB) and Stutter ratio of the 31 autosomal STR markers have been mentioned in Fig. 2. Out of all the STR markers, D4S2408 showed the most perfect average LB value (0.992) whereas, D16S539 showed greatest deviation from the ideal LB value (1.0), with an average value of 1.925. Other markers which showed a greater deviation from the ideal LB value included D18S51 (0.394), D2S1338 (0.411), D3S1358 (1.513), FGA (0.371), Penta D (0.167), Penta E (0.371), TH01 (1.708), and TPOX (1.579). With an ideal value of 1.0, STR markers showed HB value in the range of 1.031 (D8S1179) and 1.722 (TH01). Out of 31 STR markers tested, relatively higher heterozygous imbalance was observed in the D12ATA63 (1.396), D19S433 (1.307), D1S1656 (1.376), D22S1045 (1.325), and TH01 (1.722). None of the markers showed a deviation for the threshold set for the stutter ratio i.e., 1.4. The occurrence of the stutter products was observed to be highest in the number for D1S1656 and null stutter product was observed for D3S4529. The average value of stutter ratio ranged from 0.104 (D16S539) to 0.127 (D6S474). As the use of NGS technology is still at its nascent stage in the forensic DNA applications, quality issues of some STR markers need to be addressed by the kit manufacturers prior to their efficient use in routine forensic casework.

### Concordance study, allele frequency, forensic and paternity parameters

**Figure 3:**
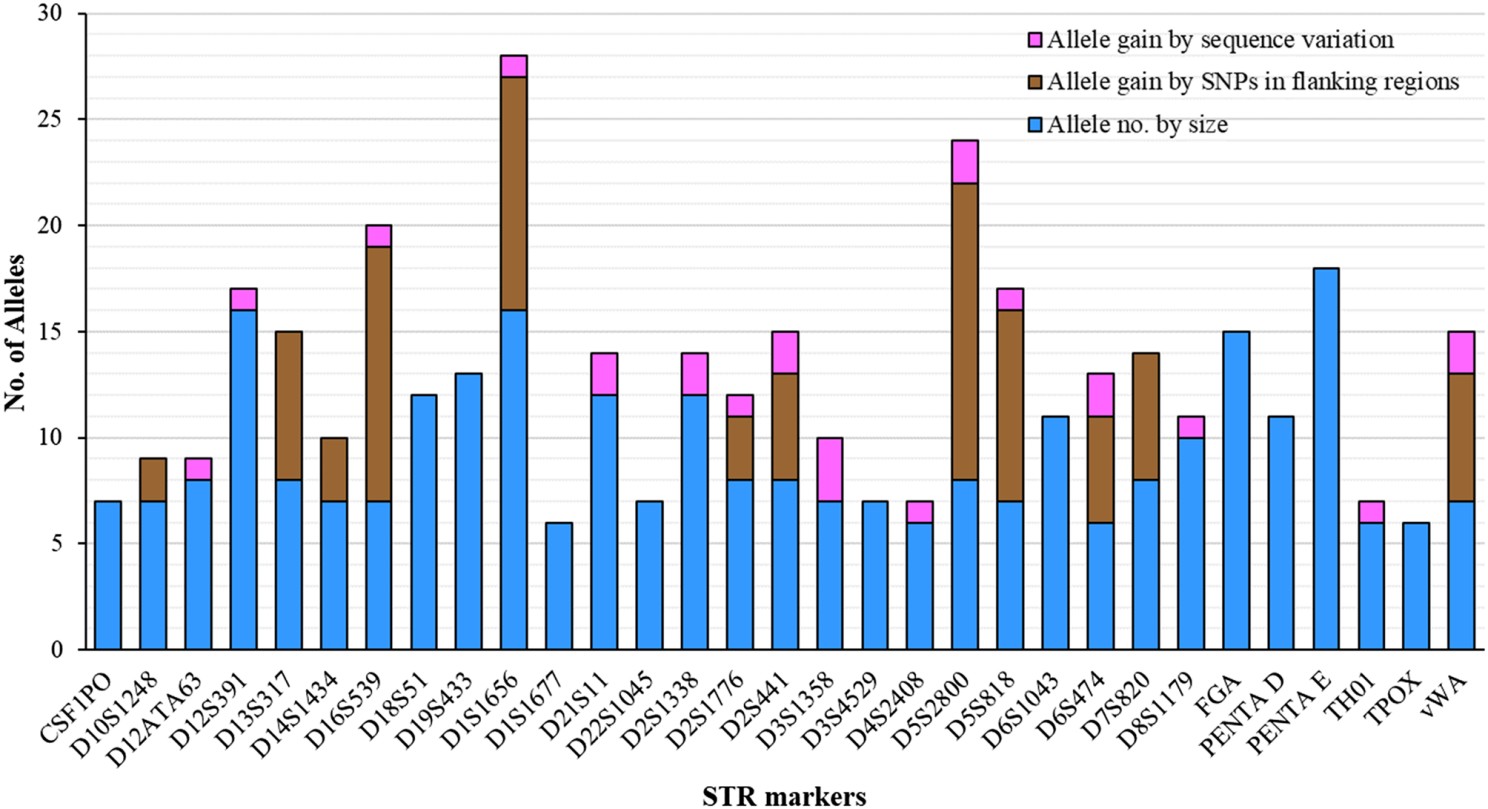
Allele gains by sequences at 31 autosomal STR markers due to SNPs in flanking regions and isometric heterozygous conditions.

Out of 31 autosomal STR markers viz. CSF1PO, D10S1248, D12ATA63, D12S391, D13S317, D14S1434, D16S539, D18S51, D19S433, D1S1656, D1S1677, D21S11, D22S1045, D2S1338, D2S1776, D2S441, D3S1358, D3S4529, D4S2408, D5S2800, D5S818, D6S1043, D6S474, D7S820, D8S1179, FGA, Penta D, Penta E, TH01, TPOX and vWA analyzed in this study; 22 overlapped STRs were compared with the length-based allele data obtained by the CE analysis. For all the samples, the length-based allele data was found to be consistent irrespective of the CE analysis or NGS data. To the best of our knowledge, this is the first report wherein sequence-based analysis of the 31 STR markers has been carried out on studied markers in any Indian population. Besides, this is also the first allelic report on nine STR markers i.e., D12ATA63, D14S1434, D1S1677, D2S1776, D3S4529, D4S2408, D5S2800, D6S1043, and D6S474 in the Indian population. The calculated length-based allele frequency values are given in the Supplementary Table S1. Forensic and paternity parameters of the length-based and sequence-based alleles have been provided in Table 1. The average total allele number of all the genetic markers was calculated as 9.26 and the highest number of size-based alleles (18) was observed on marker Penta E, whereas, D1S1677, D4S2408, and D6S474 showed the lowest number of alleles i.e., 6 (Fig. 3). The newly analyzed markers i.e., D12ATA63, D14S1434, D1S1677, D2S1776, D3S4529, D4S2408, D5S2800, D6S1043, and D6S474 generated a total allele number of 8, 7, 6, 8, 7, 6, 8, 11, and 6 respectively. Besides, Penta E showed the highest power of discrimination (0.978), polymorphic information content (0.90), Expected Heterozygosity (0.905) value, and the lowest matching probability (0.022), whereas, FGA showed the highest value for Power of Exclusion (0.778), Typical Paternity index (4.60) and observed heterozygosity (0.891). These findings suggested the usefulness of Penta E and FGA marker in the central Indian population based on the length-based analysis of alleles. D2S441 showed its least usefulness in the terms of polymorphic information content (0.64), power of exclusion (0.329), typical paternity index (1.35), observed and expected heterozygosity (0.630 and 0.690). Similarly, the calculated power of discrimination (0.855) and matching probability (0.145) values did not advocate the usefulness of the D5S818 marker in the studied population. On the contrary, when sequence-based forensic and paternity parameters were calculated in 31 autosomal STR markers, D2S1338 emerged to be the most useful marker in the studied population with the highest values of power of discrimination (0.984), polymorphic information content (0.920), power of exclusion (0.822), and typical paternity index (5.75), and the lowest matching probability (0.016). This suggested that the individual markers should be assessed on the basis of sequence-based alleles to get a clear idea on their usefulness in a specific population.

**Table 1:** Calculated forensic and paternity parameters of the 31 autosomal STR based on length-based (LB) and sequence-based (SB) alleles in the central Indian population (n = 138).

Parameters		CSF1PO	D10S1248	D12ATA63	D12S391	D13S317	D14S1434	D16S539	D18S51	D19S433	D1S1656	D1S1677	D21S11	D22S1045	D2S1338	D2S1776	D2S441	D3S1358	D3S4529	D4S2408	D5S2800	D5S818	D6S1043	D6S474	D7S820	D8S1179	FGA	PENTA D	PENTA E	TH01	TPOX	vWA
Forensic and paternity parameters																																
PD	LB	0.867	0.893	0.929	0.961	0.923	0.868	0.928	0.950	0.947	0.965	0.862	0.960	0.883	0.964	0.904	0.862	0.900	0.904	0.909	0.906	0.855	0.944	0.910	0.924	0.946	0.955	0.945	0.978	0.906	0.863	0.930
	SB	0.867	0.893	0.939	0.972	0.923	0.868	0.928	0.950	0.947	0.972	0.862	0.980	0.883	0.984	0.906	0.903	0.965	0.904	0.911	0.945	0.858	0.944	0.910	0.924	0.969	0.955	0.945	0.978	0.914	0.863	0.946
PIC	LB	0.67	0.73	0.76	0.84	0.77	0.67	0.77	0.82	0.80	0.87	0.65	0.84	0.69	0.85	0.73	0.64	0.71	0.71	0.73	0.73	0.67	0.80	0.73	0.75	0.82	0.85	0.80	0.90	0.72	0.65	0.78
	SB	0.67	0.73	0.78	0.87	0.77	0.67	0.78	0.82	0.80	0.89	0.65	0.90	0.69	0.92	0.73	0.71	0.86	0.71	0.74	0.81	0.68	0.80	0.74	0.75	0.88	0.85	0.80	0.90	0.74	0.65	0.81
PE	LB	0.456	0.662	0.567	0.676	0.516	0.456	0.554	0.594	0.594	0.748	0.358	0.567	0.411	0.719	0.516	0.329	0.400	0.422	0.491	0.580	0.554	0.607	0.467	0.479	0.748	0.778	0.529	0.763	0.479	0.452	0.634
	SB	0.456	0.662	0.580	0.748	0.516	0.456	0.594	0.594	0.594	0.807	0.358	0.662	0.411	0.822	0.529	0.422	0.691	0.422	0.529	0.676	0.580	0.607	0.491	0.479	0.792	0.778	0.529	0.763	0.504	0.452	0.676
PI	LB	1.77	3.00	2.30	3.14	2.03	1.77	2.23	2.46	2.46	4.06	1.44	2.30	1.60	3.63	2.03	1.35	1.57	1.64	1.92	2.38	2.23	2.56	1.82	1.86	4.06	4.60	2.09	4.31	1.86	1.76	2.76
	SB	1.77	3.00	2.38	4.06	2.03	1.77	2.46	2.46	2.46	5.31	1.44	3.00	1.60	5.75	2.09	1.64	3.29	1.64	2.09	3.14	2.38	2.56	1.92	1.86	4.93	4.60	2.09	4.31	1.97	1.76	3.14
Ho	LB	0.717	0.833	0.783	0.841	0.754	0.717	0.775	0.797	0.797	0.877	0.652	0.783	0.688	0.862	0.754	0.630	0.681	0.696	0.739	0.790	0.775	0.804	0.725	0.732	0.877	0.891	0.761	0.884	0.732	0.710	0.819
	SB	0.717	0.833	0.790	0.877	0.754	0.717	0.797	0.797	0.797	0.906	0.652	0.833	0.688	0.913	0.761	0.696	0.848	0.696	0.761	0.841	0.790	0.804	0.739	0.732	0.899	0.891	0.761	0.884	0.746	0.710	0.841
Pm	LB	0.133	0.107	0.071	0.039	0.077	0.132	0.072	0.050	0.053	0.035	0.138	0.040	0.117	0.036	0.096	0.138	0.100	0.096	0.091	0.094	0.145	0.056	0.090	0.076	0.054	0.045	0.055	0.022	0.094	0.137	0.070
	SB	0.133	0.107	0.061	0.028	0.077	0.132	0.072	0.050	0.053	0.028	0.138	0.020	0.117	0.016	0.094	0.097	0.035	0.096	0.089	0.055	0.142	0.056	0.090	0.076	0.031	0.045	0.055	0.022	0.086	0.137	0.054

*PD* power of discrimination, *PIC* polymorphism information content, *PE* power of exclusion, *PI* typical paternity index, *Ho* observed heterozygosity, *Pm* matching probability.

The previous studies also suggested the utility of the Penta E marker with higher forensic and paternity parameters in the Indian population^16–18^. This marker has already been established with high forensic efficiency for its effective use in the personal identification in the Portuguese population^19^, Austrian Caucasian population^20^, Northern Italy population^21^ and Mexican population^22^. When the newly inducted STR markers i.e., D12ATA63, D14S1434, D1S1677, D2S1776, D3S4529, D4S2408, D5S2800, D6S1043, and D6S474 were analyzed, they showed a similar allelic range and other statistical parameters in the limited published literature from Inner Mongolia, China^23^, Tujia population^24^.

Out of 81 male samples, four samples were found to be of AMELY deletion cases; where, AMELY could not be amplified, but a positive amplification was present in three alternative sex-determining markers i.e., DYS391, SRY, and Y InDel. This result was found to be consistent with the corresponding CE data. Allele no. 10 was found to be present dominantly in 63 samples followed by allele 11 (16 samples) and allele 9 (2 samples). Similarly, Y InDel showed allele 2 in 74 samples and allele 1 in only 7 male samples. AMELY deletion is a global problem^25^ and simultaneous amplification of the alternative sex-determining markers^26,27^ is highly useful in assigning the sex of a sample appropriately as evidenced in four samples of the current study.

### Increment in allele number by sequencing

A huge increase in the sequence-based allele number was detected in the studied STRs in comparison to the length-based allele numbers (Fig. 3). It has been previously studied that the presence of SNPs in STR flanking regions and allele sequence variation with similar length, majorly contribute to such increment in the allele numbers^28^. Substantial gain in allele numbers has been detected at D13S317, D16S539, D1S1656, D5S2800, D5S818, D7S820, and vWA with D5S2800 showing a significant increase in allele numbers due to the variation in flanking region and D3S1358 showed the highest allele gain due to the differing repeat sequence conditions. On the contrary, the genetic markers which showed no gain in allele numbers either by SNPs in flanking regions or sequence length variation included CSF1PO, D18S51, D19S433, D1S1677, D22S1045, D22S1045, D3S4529, D6S1043, FGA, Penta D, Penta E, and TPOX. Besides, the markers which showed an increment in allele number only due to SNPs in flanking regions were D10S1248, D13S317, D14S1434, and D7S820. The increased allele number in D12ATA63, D12S391, D21S11, D2S1338, D3S1358, D4S2408, D8S1179, and TH01, was due to the variation in the repeat sequences only.

Short nucleotide polymorphism (SNPs) associated with the flanking region of STRs has widely been reported throughout the globe^13,29,30^. The SNP-STR links SNPs with the STR polymorphism which allows the generation of an STR allele subtype, based on the observed SNP allele in the flanking region. Although many other marker combinations such as deletion-insertion polymorphisms amplified with STRs (DIP-STR) are used widely, a recent study advocated the use of SNP-STRs for forensic application, where an imbalanced DNA mixture is expected^31^. In this regard, the current study depicted the existence of many SNPs in the flanking region of STRs in the studied population (Table 2). rs25768 showed the highest occurrence in the central Indian population associated at upstream of D5S818 marker, whereas, rs73250432, rs369257353, and rs561924992 located at upstream of D13S317, downstream of D5S818, and downstream of D16S539 respectively showed their least occurrence.

**Table 2:** Observed SNPs and their characteristics in flanking regions of STRs in the current study.

SNPs	Chromosomal position	Most frequent allele	Least frequent allele	Frequency of least frequent allele	STR marker	Upstream/downstream
rs9546005	13q	A	T	0.196	D13S317	Downstream
rs73250432	13q	C	T	0.002		Upstream
rs25768	5q	A	G	0.265	D5S818	Upstream
rs369257353	5q	A	C	0.002		Downstream
rs16887642	7q	G	A	0.075	D7S820	Downstream
rs146350460	14q	C	T	0.004	D14S1434	Upstream
rs11642858	16q	A	C	0.122	D16S539	Downstream
rs4847015	1q	C	T	0.047	D1S1656	Downstream
rs62357468	5q	G	C	0.079	D5S2800	Upstream
rs12187142	5q	C	T	0.078		Downstream
rs74640515	2p	G	A	0.028	D2S441	Upstream
rs75219269	12p	A	G	0.049	vWA	Upstream
rs563997442	16q	C	G	0.029	D16S539	Upstream
rs561924992	16q	A	T	0.002		Downstream
rs62414880	6q	A	G	0.016	D6S474	Upstream
rs191784375	2q	C	T	0.004	D2S1776	Downstream
rs531980552	10q	C	T	0.002	D10S1248	Downstream

**Figure 4:**
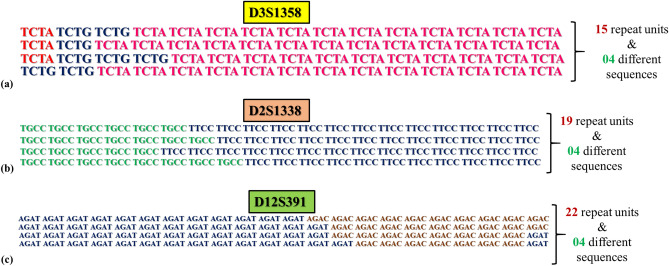
A representative image showing allele sequence variations with the same length (**a**) 15 repeats at D3S1358, (**b**) 19 repeats at D2S1338, and (**c**) 22 repeats at D12S391.

Detection of alleles with identical size but different internal sequence variation has been acknowledged as one of the advantages of using NGS for studying STRs^32,33^. The marker-wise isoalleles observed in the central Indian population have been reported in the Table S2. Out of 31 autosomal STR markers analyzed in this study, the isometric heterozygous pattern was observed at only 16 loci i.e., D3S1358, D21S11, vWA, D5S2800, D6S474, D2S441, D12ATA63, D2S1338, D1S1656, D16S539, D8S1179, D12S391, D2S1776, TH01, D5S818, and D4S2408. Allele no. 15 of D3S1358, allele no. 19 of D2S1338, and allele no. 22 of D12S391 showed a maximum number of isoalleles with the same size and different intervening sequences (Fig. 4).

A previous report has suggested a correlation between the allele number and various paternity and forensic parameters of an STR marker such as total possible genotypes, Power of discrimination, Matching probability, Polymorphic information content, power of exclusion, total paternity index, and gene diversity^18^. Keeping this in view, a substantial increase in sequence-based allele numbers in the STRs as observed in the present study increased their evidentiary value. With the increase in the allele number, the potential forensic and paternity applications of the STR markers are substantially increased. An increase in the allele number has further been correlated with the increase in heterozygosity of an STR marker which also increased its informativeness^9^.

### Population genetics

**Figure 5:**
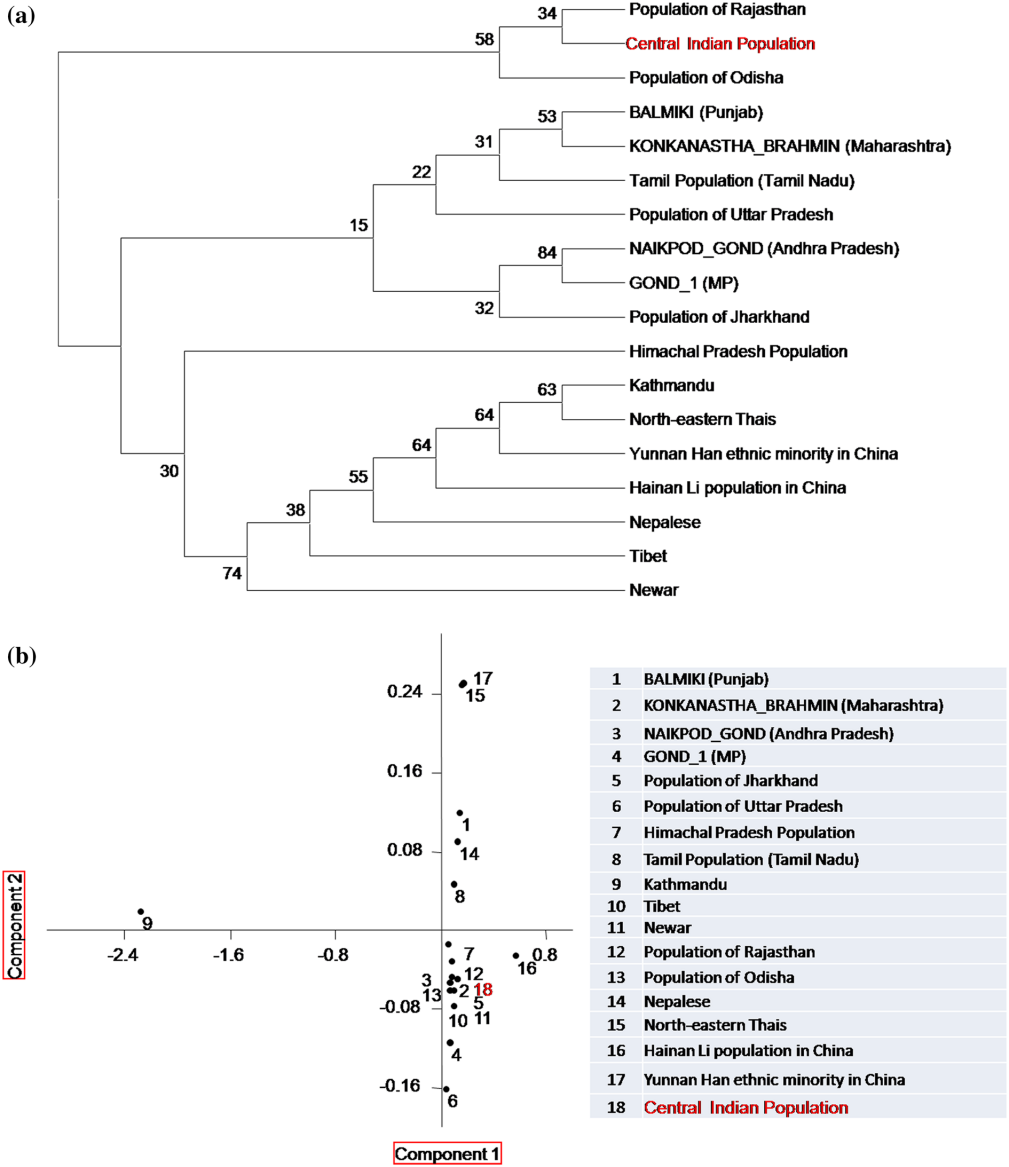
(**a**) Neighbour Joining phylogenetic tree, and (**b**) PCA plot showing relatedness of the observed size-based alleles of consistent 15 autosomal STR markers with different populations.

**Figure 6:**
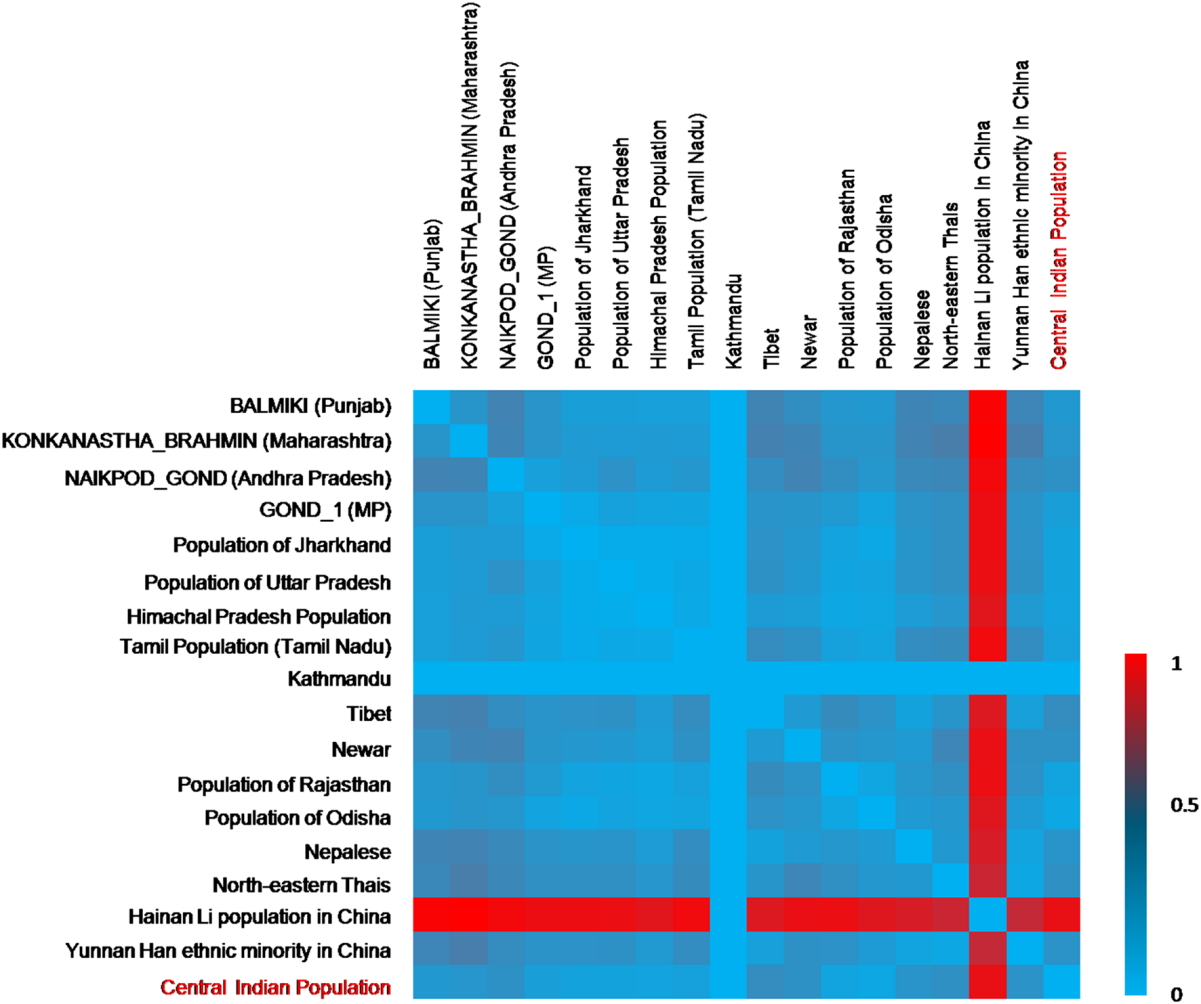
Interpopulation genetic structure at 15 consistent autosomal STR loci.

When the observed size-based allelic data were compared at 15 consistent STR markers of the different populations and a neighbor-joining tree was constructed (Fig. 5a), the dendogram showed two distinct branches of the population clusters. One cluster included the population of Tibet, Nepal, China Han population from Yunnan Province, Southwest China, northeastern Thai people of Thailand, Hainan Li population from China, Kathmanduand Newar population, Nepal. The studied Central Indian population showed a close affinity with the population of Rajasthan, India, and the population of Odisha, India. Further, a consistent result was obtained in PCA plot based on the component 1 and component 2 (Fig. 5b), where, clustering of populations from Madhya Pradesh (Gond), Jharkhand, Uttar Pradesh, Tamilnadu, Rajasthan, Himachal Pradesh and Odisha states was observed. Therefore, the genetic sharing largely mimiced the geographical clustering. The heat map drawn using Nei’s Da distance matrix has been shown in Fig. 6. The overall result of the heat map was found in concordance with the outcomes of the NJ and PCA plot for the interpopulation comparison.

## Conclusions

This first report to the best of our knowledge of sequence-based allelic data on the Central Indian population holds prominent usefulness in the forensic case works. Data obtained in this study further emphasized the implementation of NGS-based studies of STRs for forensic application. The size-based alleles showed concordance between the CE analysis as well as the NGS data. Some STR markers demonstrated a substantial variation in the repeat motifs as well as SNPs in the STR flanking regions in this study. A significant increase in the allele number further increased the statistical values of the studied forensic and paternity parameters of the STRs, thus, increasing their usefulness in the forensic applications. As per the recommendations of the ISFG, it is utmost importance to enrich the allelic data of the sequence-based STR genotypes. An increase in the allele number as evidenced in the present study also suggested the population-specific and sequence-based studies of the STR markers. In this context, the present study would be useful for providing the pioneer sequence-based data on the central Indian population.

## Materials and methods

### Sample collection and ethical statement

The current study and the experimental protocols were approved by the Ethics Committee of Banaras Hindu University, Varanasi, India (Ref. No. I.Sc./ECM-XII/2018–19/06). All the experimental procedures were carried out in accordance with the relevant guidelines and regulations laid by the ethical committee. Before the collection of the blood samples, written informed consent was obtained from each sample donor. Peripheral blood samples of 138 unrelated adult individuals consisting of 81 males and 57 females were collected in K_2_EDTA vacutainers and were stored at 4ºC till further use. Such samples were considered from the routine forensic cases at DNA Fingerprinting Unit, Forensic Science Laboratory, Bhopal, Madhya Pradesh, India and included in this study. The study was conducted following all the required quality control measures at Forensic Science Laboratory, Bhopal, M.P., India.

### DNA extraction and quantification

Genomic DNA was extracted using PrepFiler Express™ Forensic DNA Extraction Kit (Thermo Scientific, US) following the manufacturer’s guidelines. The extracted DNA was quantified using Quantifiler® Trio DNA Quantification Kit (Thermo Scientific, US) and QuantStudio™ 3 Real-Time PCR System (Thermo Scientific, US) according to the manufacturer’s instructions. Further, the concentration of DNA samples wwas adjusted to 1.0 ng/µl using TE buffer and stored at − 20 °C until further use. The authors have passed the Academia Iberoamericana de Criminalística y EstudiosForenses (AICEF) DNA Proficiency test of the de BIOLOGIA y QUÍMICA FORENSE (GITAD), Spain (http://gitad.ugr.es/principal.htm).

### Library preparation and quantitation

Genomic DNA isolated from the sample was converted to a sequencing library by targeted amplification of the regions of interest by using Precision ID DL8 kit and Precision ID GlobalFiler™ NGS STR panel v2 (Thermo Scientific, US) following manufacturer’s protocol on HID Ion Chef™ System (Thermo Scientific, US). Before that, each DNA sample was normalized to 1 ng in 15 µl volume followed by transfer of 15 µl normalized DNA into one of eight wells (A1-H1 position) of the IonCode™ Barcode Adapters plate. Subsequently, the plate with loaded DNA and other consumables was loaded at the designated places in the HID Ion Chef™ System to start the process of library preparation. The library preparation was carried out similarly for other 18 runs. Each library contained eight samples except the 18^th^ library which had only 2 samples. Once the library preparation was completed, they were stored at -20° till further use. Further, the pooled libraries were quantified on the QuantStudio 5 Real-Time PCR system using Ion Library TaqMan® Quantitation kit (Thermo Scientific, US) following the manufacturer’s recommendations.

### Template preparation, sequencing, and data analysis

Libraries that were prepared by automation were clonally amplified on the Ion Chef System by emulsion PCR of library molecules captured on the beads. The pooled libraries were diluted to 50 pM and mixed according to the group of barcode adaptors to accommodate 32 samples. 25 µl of each diluted library pool was loaded onto the Position A and Position B of the Ion S5™ Precision ID Chef Reagents along with other recommended plastic wares and reagents at the designated places onto the Ion Chef™ system. The Ion Chef System automated all template preparation steps, including creating the emulsion mixture, performing the PCR, carrying out the post-PCR purifications, and finally loading the purified templated beads onto the two Ion 530 chips accordingly using the manufacturer’s guidelines.

### Sequencing

A sequencing run on the Ion S5 systems was initiated by loading a reagent cartridge, buffer, cleaning solution, and waste container as per the Ion S5™ Precision ID Sequencing Kit protocol of the manufacturer. The Ion S5 chip was then loaded and the run started using 200 bp chemistry with 650 flow according to the human identification GlobalFiler™ NGS STR sequencing format.

The raw data was extracted from the S5 Torrent Server v5.10.0 (Thermo Fisher Scientific) and were input into the Converge™ software v2.1 (Thermo Fisher Scientific) for sequence analysis with *Homo sapiens* hg19 genome. The HID Genotyper plugin v2.1 (Thermo Fisher Scientific) was applied to the analysis procedure at the default thresholds, in which the relative analytical and stochastic thresholds were both 0.05 and the stutter ratio was set as 0.14. Further sequencing performance of Precision ID NGS STR panel v2 was assessed by analyzing locus balance (LB), heterozygous balance (HB), and stutter ratio of the obtained sequences following Avila et al.^34^ and Brookes et al.^35^.

### Concordance analysis with capillary electrophoresis (CE)

All the 138 samples were studied to assess the concordance between CE-STR data and NGS-STR data. All these samples were analyzed using the PowerPlex Fusion 6C System (Promega, USA) following the manufacturer’s guidelines. 0.5–1.0 ng of genomic DNA was used to amplify the samples on Veriti 96 well Thermal Cycler (Thermo Scientific, USA). Capillary electrophoresis of the amplified DNA fragments was performed using a 3500xL Genetic Analyzer (Thermo Scientific, USA). The generated STR fragments were analyzed using GeneMapper ID-X v.1.5 software maintaining a threshold of 200 RFU for all the dye sets. The CE based allelic values were compared with the sequencing-based allelic values at 23 consistent loci between Fusion 6C System and GlobalFiler NGS STR panel i.e., CSF1PO, D10S1248, D12S391, D13S317, D16S539, D18S51, D19S433, D1S1656, D21S11, D22S1045, D2S1338, D2S441, D3S1358, D5S818, D7S820, D8S1179, DYS391, FGA, Penta D, Penta E, TH01, TPOX, vWA, and sex determining marker Amelogenin.

### Statistical analysis

Obtained sequence and allele data were evaluated for the presence of isometric heterozygous alleles and the presence/absence of SNPs in the flanking regions. Besides, various forensic and paternity parameters such as Allele frequency, Power of Discrimination (PD), Polymorphism information content (PIC), Power of exclusion (PE), Typical paternity index (PI), Observed (Ho), Matching Probability (Pm) were calculated using GenAlEx 6.5 software^36^, Arlequin v3.5 software^37^ and AMOVA for both length-based and sequence-based alleles. The observed size-based allele frequencies of the 15 consistent genetic markers were compared with the data obtained in the previously published literature by using the Fst pairwise distance.

The compared allele frequency data of the published populations included Balmiki population, Punjab, India^38^, Konkanastha Brahmin population, Maharashtra, India^38^, Naikpod Gond, Andhra Pradesh, India^39^, Gond, Madhya Pradesh, India^40^, Population of Jharkhand, India^41^, Populations of Uttar Pradesh, India^42^, population of Himachal Pradesh, India^43^, Tamil population, Tamil Nadu, India^44^, Tibetan population, Nepal^45^, population of Newar, Nepal^46^, population of Rajasthan, India^47^, population of Odisha, India^48^, Nepalese population^49^_,_ Chinese Han population from Yunnan Province, Southwest China^50^, northeastern Thai people of Thailand^51^ and Hainan Li population from China^52^.

## Supplementary Information

Supplementary Tables.
