# Supplementary material for: Sequence variations, flanking region mutations, and allele frequency at 31 autosomal STRs in the central Indian population by next generation sequencing (NGS)
Source: Sci Rep. 2021 Dec 1;11:23238. doi: 10.1038/s41598-021-02690-5 (PMC8636586; doi:10.1038/s41598-021-02690-5)
Supplement: Supplementary file 1 — Supplementary Tables. [file 41598_2021_2690_MOESM1_ESM.docx]

**Sequence variations, flanking region mutations, and allele frequency at 31 autosomal STRs in the Central Indian population by Next Generation Sequencing (NGS)**

**Table S1.** Length-based allele frequency distribution in the central Indian population (N=138)

| **Alleles** | **CSF1PO** | **D10S1248** | **D12ATA63** | **D12S391** | **D13S317** | **D14S1434** | **D16S539** | **D18S51** | **D19S433** | **D1S1656** | **D1S1677** | **D21S11** | **D22S1045** | **D2S1338** | **D2S1776** | **D2S441** | **D3S1358** | **D3S4529** | **D4S2408** | **D5S2800** | **D5S818** | **D6S1043** | **D6S474** | **D7S820** | **D8S1179** | **FGA** | **PENTA D** | **PENTA E** | **TH01** | **TPOX** | **vWA** |
| --- | --- | --- | --- | --- | --- | --- | --- | --- | --- | --- | --- | --- | --- | --- | --- | --- | --- | --- | --- | --- | --- | --- | --- | --- | --- | --- | --- | --- | --- | --- | --- |
| **4** |  |  |  |  |  |  |  |  |  |  |  |  |  |  |  |  |  |  |  |  | 0.004 |  |  |  |  |  |  |  |  |  |  |
| **5** |  |  |  |  |  |  |  |  |  |  |  |  |  |  |  |  |  |  |  |  |  |  |  |  |  |  |  | 0.051 |  |  |  |
| **6** |  |  |  |  |  |  |  |  |  |  |  |  |  |  |  |  |  |  |  |  |  |  |  |  |  |  | 0.007 |  | 0.207 |  |  |
| **7** |  |  |  |  | 0.004 |  |  |  |  |  |  |  |  |  |  |  |  |  |  |  |  |  |  | 0.011 |  |  | 0.014 | 0.058 | 0.174 |  |  |
| **8** |  |  |  | 0.004 | 0.243 |  | 0.080 |  |  | 0.029 |  |  |  |  | 0.047 |  |  |  | 0.304 |  |  |  |  | 0.243 |  |  | 0.011 | 0.007 | 0.130 | 0.344 |  |
| **9** | 0.025 |  |  |  | 0.076 |  | 0.130 |  |  |  |  |  |  |  | 0.036 |  |  |  | 0.232 |  | 0.014 | 0.007 |  | 0.051 | 0.004 |  | 0.228 | 0.018 | 0.377 | 0.138 |  |
| **9.3** |  |  |  |  |  |  |  |  |  |  |  |  |  |  |  |  |  |  |  |  |  |  |  |  |  |  |  |  | 0.098 |  |  |
| **10** | 0.178 |  |  |  | 0.109 | 0.170 | 0.094 | 0.004 |  | 0.004 |  |  |  |  | 0.109 | 0.312 |  |  | 0.214 |  | 0.127 | 0.011 |  | 0.250 | 0.188 | 0.004 | 0.167 | 0.029 | 0.014 | 0.091 |  |
| **10.3** |  |  |  |  |  |  |  |  |  |  |  |  |  |  |  |  |  |  |  |  |  |  |  | 0.007 |  |  |  |  |  |  |  |
| **11** | 0.355 | 0.011 |  |  | 0.221 | 0.062 | 0.301 | 0.036 | 0.007 | 0.156 | 0.004 |  | 0.239 |  | 0.268 | 0.442 |  | 0.004 | 0.181 |  | 0.391 | 0.286 |  | 0.228 | 0.072 |  | 0.246 | 0.192 |  | 0.388 |  |
| **11.3** |  |  |  |  |  |  |  |  |  |  |  |  |  |  |  | 0.054 |  |  |  |  |  |  |  |  |  |  |  |  |  |  |  |
| **11.4** |  |  |  |  |  |  |  |  |  |  |  |  |  |  |  |  |  |  |  |  |  |  |  |  |  |  | 0.004 |  |  |  |  |
| **12** | 0.344 | 0.022 | 0.330 |  | 0.275 | 0.040 | 0.239 | 0.069 | 0.087 | 0.072 | 0.051 |  | 0.004 |  | 0.362 | 0.040 | 0.004 | 0.011 | 0.065 |  | 0.297 | 0.239 |  | 0.188 | 0.127 |  | 0.141 | 0.076 |  | 0.036 |  |
| **12.2** |  |  |  |  |  |  |  |  | 0.018 |  |  |  |  |  |  |  |  |  |  |  |  |  |  |  |  |  |  |  |  |  |  |
| **13** | 0.087 | 0.127 | 0.047 |  | 0.051 | 0.250 | 0.134 | 0.116 | 0.293 | 0.130 | 0.134 |  |  |  | 0.141 | 0.025 |  | 0.290 | 0.004 |  | 0.152 | 0.098 |  | 0.022 | 0.159 |  | 0.120 | 0.062 |  | 0.004 |  |
| **13.2** |  |  |  |  |  |  |  |  | 0.022 |  |  |  |  |  |  |  |  |  |  |  |  |  |  |  |  |  |  |  |  |  |  |
| **14** | 0.004 | 0.221 | 0.062 |  | 0.022 | 0.431 | 0.022 | 0.272 | 0.250 | 0.138 | 0.348 |  | 0.065 | 0.004 | 0.033 | 0.112 | 0.043 | 0.279 |  | 0.268 | 0.014 | 0.069 | 0.228 |  | 0.203 |  | 0.043 | 0.072 |  |  | 0.109 |
| **14.2** |  |  |  |  |  |  |  |  | 0.076 |  |  |  |  |  |  |  |  |  |  |  |  |  |  |  |  |  |  |  |  |  |  |
| **15** | 0.007 | 0.351 | 0.156 | 0.011 |  | 0.043 |  | 0.170 | 0.101 | 0.145 | 0.391 |  | 0.395 |  | 0.004 | 0.011 | 0.304 | 0.250 |  |  |  |  | 0.333 |  | 0.170 |  | 0.018 | 0.087 |  |  | 0.083 |
| **15.2** |  |  |  |  |  |  |  |  | 0.065 |  |  |  |  |  |  |  |  |  |  |  |  |  |  |  |  |  |  |  |  |  |  |
| **15.3** |  |  |  |  |  |  |  |  |  | 0.018 |  |  |  |  |  |  |  |  |  |  |  |  |  |  |  |  |  |  |  |  |  |
| **16** |  | 0.207 | 0.083 | 0.007 |  | 0.004 |  | 0.170 | 0.051 | 0.163 | 0.072 |  | 0.207 | 0.007 |  | 0.004 | 0.279 | 0.138 |  | 0.043 |  |  | 0.149 |  | 0.058 | 0.004 |  | 0.116 |  |  | 0.217 |
| **16.2** |  |  |  |  |  |  |  |  | 0.018 |  |  |  |  |  |  |  |  |  |  |  |  |  |  |  |  |  |  |  |  |  |  |
| **16.3** |  |  |  |  |  |  |  |  |  | 0.018 |  |  |  |  |  |  |  |  |  |  |  |  |  |  |  |  |  |  |  |  |  |
| **17** |  | 0.062 | 0.243 | 0.141 |  |  |  | 0.083 | 0.007 | 0.062 |  |  | 0.080 | 0.022 |  |  | 0.257 | 0.029 |  | 0.163 |  | 0.022 | 0.196 |  | 0.007 |  |  | 0.098 |  |  | 0.286 |
| **17.2** |  |  |  |  |  |  |  |  | 0.004 |  |  |  |  |  |  |  |  |  |  |  |  |  |  |  |  |  |  |  |  |  |  |
| **17.3** |  |  |  | 0.007 |  |  |  |  |  | 0.051 |  |  |  |  |  |  |  |  |  |  |  |  |  |  |  |  |  |  |  |  |  |
| **18** |  |  | 0.072 | 0.257 |  |  |  | 0.033 |  | 0.004 |  |  |  | 0.149 |  |  | 0.105 |  |  | 0.351 |  | 0.120 | 0.087 |  | 0.011 | 0.004 |  | 0.062 |  |  | 0.185 |
| **18.2** |  |  |  |  |  |  |  |  |  | 0.004 |  |  |  |  |  |  |  |  |  |  |  |  |  |  |  |  |  |  |  |  |  |
| **18.3** |  |  |  | 0.011 |  |  |  |  |  |  |  |  |  |  |  |  |  |  |  |  |  |  |  |  |  |  |  |  |  |  |  |
| **19** |  |  | 0.007 | 0.163 |  |  |  | 0.025 |  | 0.004 |  |  | 0.011 | 0.199 |  |  | 0.007 |  |  | 0.007 |  | 0.076 | 0.007 |  |  | 0.087 |  | 0.025 |  |  | 0.116 |
| **19.2** |  |  |  | 0.004 |  |  |  |  |  |  |  |  |  |  |  |  |  |  |  |  |  |  |  |  |  |  |  |  |  |  |  |
| **19.3** |  |  |  |  |  |  |  |  |  | 0.004 |  |  |  |  |  |  |  |  |  |  |  |  |  |  |  |  |  |  |  |  |  |
| **20** |  |  |  | 0.123 |  |  |  | 0.014 |  |  |  |  |  | 0.109 |  |  |  |  |  | 0.076 |  | 0.069 |  |  |  | 0.120 |  | 0.033 |  |  | 0.004 |
| **20.2** |  |  |  |  |  |  |  |  |  |  |  |  |  |  |  |  |  |  |  |  |  |  |  |  |  | 0.004 |  |  |  |  |  |
| **21** |  |  |  | 0.058 |  |  |  |  |  |  |  |  |  | 0.047 |  |  |  |  |  |  |  | 0.004 |  |  |  | 0.091 |  |  |  |  |  |
| **21.2** |  |  |  |  |  |  |  |  |  |  |  |  |  |  |  |  |  |  |  |  |  |  |  |  |  | 0.007 |  |  |  |  |  |
| **22** |  |  |  | 0.098 |  |  |  | 0.007 |  |  |  |  |  | 0.094 |  |  |  |  |  |  |  |  |  |  |  | 0.196 |  | 0.007 |  |  |  |
| **22.2** |  |  |  |  |  |  |  |  |  |  |  |  |  |  |  |  |  |  |  |  |  |  |  |  |  | 0.011 |  |  |  |  |  |
| **23** |  |  |  | 0.072 |  |  |  |  |  |  |  |  |  | 0.156 |  |  |  |  |  | 0.058 |  |  |  |  |  | 0.181 |  | 0.004 |  |  |  |
| **24** |  |  |  | 0.025 |  |  |  |  |  |  |  |  |  | 0.141 |  |  |  |  |  | 0.033 |  |  |  |  |  | 0.174 |  |  |  |  |  |
| **25** |  |  |  | 0.014 |  |  |  |  |  |  |  |  |  | 0.062 |  |  |  |  |  |  |  |  |  |  |  | 0.080 |  | 0.004 |  |  |  |
| **26** |  |  |  | 0.004 |  |  |  |  |  |  |  |  |  | 0.011 |  |  |  |  |  |  |  |  |  |  |  | 0.029 |  |  |  |  |  |
| **27** |  |  |  |  |  |  |  |  |  |  |  | 0.018 |  |  |  |  |  |  |  |  |  |  |  |  |  | 0.011 |  |  |  |  |  |
| **28** |  |  |  |  |  |  |  |  |  |  |  | 0.170 |  |  |  |  |  |  |  |  |  |  |  |  |  |  |  |  |  |  |  |
| **29** |  |  |  |  |  |  |  |  |  |  |  | 0.152 |  |  |  |  |  |  |  |  |  |  |  |  |  |  |  |  |  |  |  |
| **29.2** |  |  |  |  |  |  |  |  |  |  |  | 0.004 |  |  |  |  |  |  |  |  |  |  |  |  |  |  |  |  |  |  |  |
| **30** |  |  |  |  |  |  |  |  |  |  |  | 0.207 |  |  |  |  |  |  |  |  |  |  |  |  |  |  |  |  |  |  |  |
| **30.2** |  |  |  |  |  |  |  |  |  |  |  | 0.036 |  |  |  |  |  |  |  |  |  |  |  |  |  |  |  |  |  |  |  |
| **31** |  |  |  |  |  |  |  |  |  |  |  | 0.025 |  |  |  |  |  |  |  |  |  |  |  |  |  |  |  |  |  |  |  |
| **31.2** |  |  |  |  |  |  |  |  |  |  |  | 0.101 |  |  |  |  |  |  |  |  |  |  |  |  |  |  |  |  |  |  |  |
| **32.2** |  |  |  |  |  |  |  |  |  |  |  | 0.174 |  |  |  |  |  |  |  |  |  |  |  |  |  |  |  |  |  |  |  |
| **33.2** |  |  |  |  |  |  |  |  |  |  |  | 0.091 |  |  |  |  |  |  |  |  |  |  |  |  |  |  |  |  |  |  |  |
| **34.2** |  |  |  |  |  |  |  |  |  |  |  | 0.014 |  |  |  |  |  |  |  |  |  |  |  |  |  |  |  |  |  |  |  |
| **35.2** |  |  |  |  |  |  |  |  |  |  |  | 0.007 |  |  |  |  |  |  |  |  |  |  |  |  |  |  |  |  |  |  |  |

**Table S2.** List of Isoalleles observed in the present study in the central Indian population

| **STR Marker** | **Chromosomal Location** | **Allele** | **No. of variants observed** | **Variant Sequence** |
| --- | --- | --- | --- | --- |
| D3S1358 | 3p21.31 | 15 | 4 | [TCTA]1[TCTG]2[TCTA]12 |
|  |  |  |  | [TCTA]1[TCTG]1[TCTA]13 |
|  |  |  |  | [TCTA]1[TCTG]3[TCTA]11 |
|  |  |  |  | [TCTG]2[TCTA]13 |
|  |  | 16 | 3 | [TCTA]1[TCTG]3[TCTA]12 |
|  |  |  |  | [TCTA]1[TCTG]2[TCTA]13 |
|  |  |  |  | [TCTA]1[TCTG]1[TCTA]14 |
|  |  | 17 | 2 | [TCTA]1[TCTG]2[TCTA]14 |
|  |  |  |  | [TCTA]1[TCTG]3[TCTA]13 |
| D21S11 | 21q21.1 | 28 | 2 | [TCTA]4[TCTG]6[TCTA]3TA[TCTA]3TCA[TCTA]2TCCATA[TCTA]10 |
|  |  |  |  | [TCTA]4[TCTG]5[TCTA]3TA[TCTA]3TCA[TCTA]2TCCATA[TCTA]11 |
|  |  | 30 | 2 | [TCTA]4 [TCTG]6 [TCTA]3 TA [TCTA]3 TCA [TCTA]2 TCCATA [TCTA]12 |
|  |  |  |  | [TCTA]6 [TCTG]5 [TCTA]3 TA [TCTA]3 TCA [TCTA]2 TCCATA [TCTA]11 |
| vWA | 12p13.31 | 15 | 2 | [TCTA]1 [TCTG]3 [TCTA]11 |
|  |  |  |  | [TCTA]1 [TCTG]4 [TCTA]10 |
|  |  | 17 | 3 | [TCTA]1[TCTG]5[TCTA]11 |
|  |  |  |  | [TCTA]1[TCTG]4[TCTA]12 |
|  |  |  |  | [TCTA]1 [TCTG]4 TCCA[TCTA]11 |
| D5S2800 | 5q11.2 | 17 | 2 | [GGTA]3[GACA]8[GATA]3[GATT]3 |
|  |  |  |  | [GGTA]3[GACA]9[GATA]2[GATT]3 |
|  |  | 18 | 2 | [GGTA]3[GACA]10[GATA]2[GATT]3 |
|  |  |  |  | [GGTA]3[GACA]9[GATA]3[GATT]3 |
| D6S474 | 6q21-22 | 14 | 2 | [AGAT]5 [GATA]9 |
|  |  |  |  | [AGAT]4 [GATA]10 |
|  |  | 16 | 2 | [AGAT]5[GATA]11 |
|  |  |  |  | [AGAT]6[GATA]10 |
| D2S441 | 2p14 | 10 | 2 | [TCTA]10 |
|  |  |  |  | [TCTA]8 TCTG[TCTA]1 |
|  |  | 11 | 2 | [TCTA]9TCTG[TCTA]1 |
|  |  |  |  | [TCTA]11 |
| D12ATA63 | 12q23.3 | 15 | 2 | [TAA]12[CAA]3 |
|  |  |  |  | [TAA]11[CAA]4 |
| D2S1338 | 2q35 | 19 | 4 | [TGCC]6[TTCC]13 |
|  |  |  |  | [TGCC]7[TTCC]12 |
|  |  |  |  | [TGCC]5 [TTCC]14 |
|  |  |  |  | [TGCC]8 [TTCC]11 |
|  |  | 24 | 2 | [TGCC]7 [TTCC]14 GTCC[TTCC]2 |
|  |  |  |  | [TGCC]8 [TTCC]13 GTCC[TTCC]2 |
| D1S1656 | 1q42 | 14 | 2 | [TAGA]13 [TAGG]1 |
|  |  |  |  | [TAGA]14 |
| D16S539 | 16q24.1 | 11 | 2 | [GATA]11 |
|  |  |  |  | [GATA]5GACA[GATA]5 |
| D8S1179 | 8q24.13 | 14 | 2 | [TCTA]14 |
|  |  |  |  | [TCTA]2TCTG[TCTA]11 |
| D12S391 | 12p13.2 | 18 | 2 | [AGAT]11 [AGAC]6 [AGAT]1 |
|  |  |  |  | [AGAT]10 [AGAC]7 [AGAT]1 |
|  |  | 22 | 4 | [AGAT]12 [AGAC]10 |
|  |  |  |  | [AGAT]13 [AGAC]9 |
|  |  |  |  | [AGAT]13[AGAC]8[AGAT]1 |
|  |  |  |  | [AGAT]14[AGAC]7[AGAT]1 |
| D2S1776 | 2q24,2q31.1 | 11 | 2 | [AGAT]11 |
|  |  |  |  | [AGAT]10AAAT |
| TH01 | 11p15.5 | 9 | 2 | [TCAT]9 |
|  |  |  |  | [TCAT]8 TCAC |
| D5S818 | 5q23.2 | 11 | 2 | [AGAT]11 |
|  |  |  |  | [AGAT]10 AGAC |
| D4S2408 | 4p15.1 | 9 | 2 | [ATCT]1 GTCT[ATCT]7 |
|  |  |  |  | [ATCT]9 |
